# Identification and Validation of the Immune Regulator CXCR4 as a Novel Promising Target for Gastric Cancer

**DOI:** 10.3389/fimmu.2021.702615

**Published:** 2021-07-12

**Authors:** Shuai Xue, Ming Ma, Songhua Bei, Fan Li, Chenqu Wu, Huanqing Li, Yanling Hu, Xiaohong Zhang, YanQing Qian, Zhe Qin, Jun Jiang, Li Feng

**Affiliations:** ^1^ Endoscopy Center, Minhang Hospital, Fudan University, Shanghai, China; ^2^ Department of Gastroenterology, Minhang Hospital, Fudan University, Shanghai, China; ^3^ Institute of Fudan Minhang Academic Health System, Minhang Hospital, Fudan University, Shanghai, China

**Keywords:** gastric cancer, immunoregulatory factors, bioinformatics analysis, CXCR4, prognosis

## Abstract

Immune checkpoint blockade has attracted a lot of attention in the treatment of human malignant tumors. We are trying to establish a prognostic model of gastric cancer (GC) based on the expression profile of immunoregulatory factor-related genes. Based on the TCGA database, we identified 234 differentially expressed immunoregulatory factors. Gene Ontology (GO) and Kyoto Encyclopedia of Genes and Genomes (KEGG) conducted enrichment analysis to clarify the biological functions of differential expression of immunoregulatory factors. STRING database predicted the interaction network between 234 differently expressed immune regulatory factors. The expression of 11 immunoregulatory factors was significantly related to the overall survival of gastric cancer patients. Univariate Cox regression analysis, Kaplan–Meier analysis and multivariate Cox regression analysis found that immunomodulatory factors were involved in the progression of gastric cancer and promising biomarkers for predicting prognosis. Among them, CXCR4 was related to the low survival of GC patients and a key immunomodulatory factor in GC. Based on TCGA data, the high expression of CXCR4 in GC was positively correlated with the advanced stage and grade of gastric cancer and related to poor prognosis. Univariate analysis and multivariate analysis indicated that CXCR4 was an independent prognostic indicator for TCGA gastric cancer patients. *In vitro* functional studies had shown that CXCR4 promoted the proliferation, migration, and invasion of gastric cancer cells. In summary, this study has determined the prognostic value of 11 immunomodulatory factors in gastric cancer. CXCR4 is an independent prognostic indicator for gastric cancer patients, which may help to improve the individualized prognostic prediction of GC and provide candidates for the diagnosis and treatment of GC.

## Introduction

As one of the widely occurred carcinomas, gastric cancer (GC) is the third primary inducer of mortality amid cancers worldwide (1). Despite the occurrence rate of GC has fallen sharply in western countries, it remains high in East Asian countries (2, 3). Nevertheless, this increasing trend of GC has decreased recently, especially the proportion of early GC cases. Currently, surgical resection is the possibly available strategy for GC, whereas it is only applied in stage I of early GC cases. Clinical stage II or stage III patients require multidisciplinary adjunctive approaches (4, 5). The primary contributor to the failure of GC treatment is drug resistance (6, 7). In the past few decades, several pivotal regulators are reported to participate in GC’s pathogenesis (8, 9). For example, METTL3-mediated m^6^A methylation of SPHK2 targets KLF2, thus promoting advanced GC (10). Human CCR4 and CAF1 deacetylase mediate the regulation of human GC cell proliferation and tumorigenicity *via* modulating the cell cycle process (11). Understanding the regulatory mechanism of GC will offer new insights into treating GC (12).

In the past decade, immune checkpoint blockade has attracted a lot of attention in the human malignant neoplasms treatment, lung carcinoma, breast carcinoma and stomach carcinoma included (13–15). In GC, several anti-PD1 therapies have been approved for GC treatment. For instance, pembrolizumab largely extends the over survival (OS) and presents increasing benefits in GC patients as the PD-L1 score increased (16–18). Herein, pembrolizumab is approved for the third-line therapy of PDL1- positive (CPS ≥1) GC (19, 20). In addition, regarding the first-line therapy of HER2-negative GC patients with PD-L1 CPS no less than 5, chemotherapy along with nivolumab becomes a newly produced treatment. Nevertheless, the regulatory mechanism of immunoregulatory factors on GC still stays unclear. Previously, several immunoregulatory factors are reported to exhibit importance in GC (21–23). For example, BICC1 is shown to be a split-new prognostic indicator for GC related to immune infiltration (24).

Researches have revealed that immune regulatory factors exhibit a relationship with the poorly prognostic status of GC patients, and promote the malignant phenotype of GC cells (25, 26). Here, our purpose is to comprehensively study the expression features and clinicopathological parameters of immunomodulatory factors, so as to uncover prospective targets in treating GC. Besides, we perform loss of function tests to confirm our bioinformatics findings. We hope that this study can provide new therapeutic targets for GC.

## Materials and Method

### Data Collection

The RNA-Seq transcriptome data cohort (STAD) and clinical or prognostic details of GC were derived from TCGA (https://cancergenome.nih.gov/). CBIORTAL (www.cbioportal.org) was employed to detect the changes in the CXCR4 genome. We acquired CXCR4 mRNA expression profile from the International Cancer Genome Collaboration Group (ICGC) and Genome-wide Pan Cancer Analysis (PCAWG).

### Selection of Immunomodulators

Currently, 10 genes (NRP1, CXCR4, METTL14, BCL11B, ZC3H13, HNMT, ASGR2, EZH2, ANXA5 and CDH2) are considered as classic immunomodulators. Here, we discovered three new immunomodulatory genes (BASP1, OsbPL1A and CD59). We further obtained the expression profiles of these identified genes from the TCGA STAD cohort with clinical details. The differential expressions of these genes in GC were shown by the Violet curve.

### Consistent Cluster Analysis

In order to further explore the immunomodulatory factors, we applied consensus cluster analysis in the STAD cohort based on immunomodulatory factors. We identified two subgroups in this cohort. Besides, we carried out gene ontology (GO) and Kyoto Encyclopedia of Genes and Genomes (KEGG) analysis to evaluate their involved functions and pathways in the light of the gene profiles in the two subgroups.

### Predictive Signature Generation

We employed the univariate Cox regression model to determine the correlation of immunoregulatory genes with the OS of GC patients. We defined it as the protection and hazard of these genes with a hazard ratio HRs <1 and HRs >1, respectively. Five genetic risk signals (NRP1, ZC3H13, CXCR4, ASGR2 and CXCR4) were determined according to the minimum standard. Besides, we calculated the risk score in view of the coefficients in the Lasso algorithm. On the basis of the average value of the risk score, we classified the TCGA STAD cohort into high-risk and low-risk groups.

### Genome Changes and Identification of Co-Expressed Genes

We applied the CBioPortal tool (http://cbioportal.org) to analyze the mutations, copy number variation (CNV) and CXCR4 mRNA changes in GC. Oncoprint provided an overall outline of the changes of CXCR4 in STAD samples. The Linkedomics platform (27) was utilized to conduct co-expression analysis. We predicted potential functions through overexpression enrichment analysis (ORA) on the basis of GO, KEGG with Reactome pathways.

### The Prognostic Value Assessment of Genetic Markers

We employed chi-square test and heat map analysis to determine clinicopathological features (age, gender, grade and stage, and survival status) in high-risk and low-risk groups. We utilized Kaplan–Meier analysis and the Log-Rank test to calculate risk scores in high-risk groups and patients with low score group OS of distinct groups. Receiving the operating characteristic (ROC) and a curve were taken to investigate the prognostic value of the patient’s survival prediction. We conducted univariate and multivariate Cox regression analysis to determine the impacts of risk score on GC prognosis.

### Cell Culture and Transfection

HFE-145, MGC-803, HGC-27, AGS, SGC-7901 and BGC-823 were acquired from the Cell Bank of the Chinese Academy of Sciences (Shanghai, China). All cells were cultured in DMEM (Gibco, USA) with 10% FBS (Gibco, USA) and 1% penicillin/streptomycin. CXCR4 knockout plasmid was ordered from Dharmacon (CA, USA). The small interference RNA (siRNA) sequence was listed below: si-CXCR4-1, GATGCCGTGGCAAACTGGTACTTTG; si-CXCR4-2, TGGTTGGCCTTATCCTGCCTGGTAT; si-NC, UUCUCCGAACGUGUCACGUTT. The full-length CXCR4 cDNA was inserted into the pcDNA3.1 vector (Invitrogen, USA). About 2 μg of overexpression plasmid or 1.5 μg of siRNA was separately transfected into 1 × 10^6^ cells in a 6 cm petri dish using 12 μl of Lipofectamine^®^2000 reagent (Invitrogen) as instruction described.

### Cell Proliferation Assay

The ability of cells to proliferate in GC cells was determined using the CCK-8 kit (Dojindo, Japan). Specified GC cells were inoculated in a 96-well plate and then treated differently at the specified time. The OD values of 450 nm were detected after incubation with CCK-8 solution on a Fluoroskan Ascent fluorometer (Thermo Fisher, Finland).

### Transwell Assay

An 8 μm Transwell chamber (Corning, USA) was set in a 24-well plate to perform the invasion assay. We plated 200 μl of GC cells in the upper chamber pre-coated with Matrigel (BD, USA). The lower chamber was filled with a complete medium. At 24 h post-incubation, we fixed the chamber with 4% paraformaldehyde and stained it in 0.1% crystal violet solution. Then, we calculated the number of samples in each group under a microscope. We conducted three independent experiments in triplicate one time. The Transwell migration assay was performed as described above but without the Matrigel.

### RNA Extraction and RT-qPCR

We employed RNeasy reagent (Qiagen, Germany) to harvest the whole RNA. RT-qPCR was conducted with SYBR Premix ex TAG Mastermix kit (Takara, Japan) on the ICycler real-time system (Bio-Rad Laboratories, USA) as manual described. Glyceraldehyde-3-phosphate dehydrogenase was an internal control. The relative RNA expression was analyzed by the 2^−ΔΔCt^ approach and presented as the target gene/internal control ratio [2^−ΔΔCt (target gene-internal control)^] (28). The data were obtained from three independent experiments in triplicate one time. The primers of CXCR4 are 5’-ACTACACCGAGGAAATGGGCT-3’ (F) and 5’-CCCACAATGCCAGTTAAGAAGA-3’ (R). The primers of GAPDH are 5’-CTGGGCTACACTGAGCACC-3’ (F) and 5’-CTGGGCTACACTGAGCACC-3’ (R).

### Statistical Analysis

All derived data were analyzed by GraphPad Prism 8.0 (GraphPad, Inc., USA) and Image-Pro Plus 6.0 and shown as the mean ± standard deviation (SD). We employed Student’s t-test and one-way analysis of variance (ANOVA) to analyze the differences existing in two groups and more groups, respectively. Kaplan–Meier method and Log-rank test were taken to plot the survival curve. *P <*0.05 meant that there was significant difference in compared groups.

## Results

### Immunoregulatory Factors Expression Features

**Figure 1:**
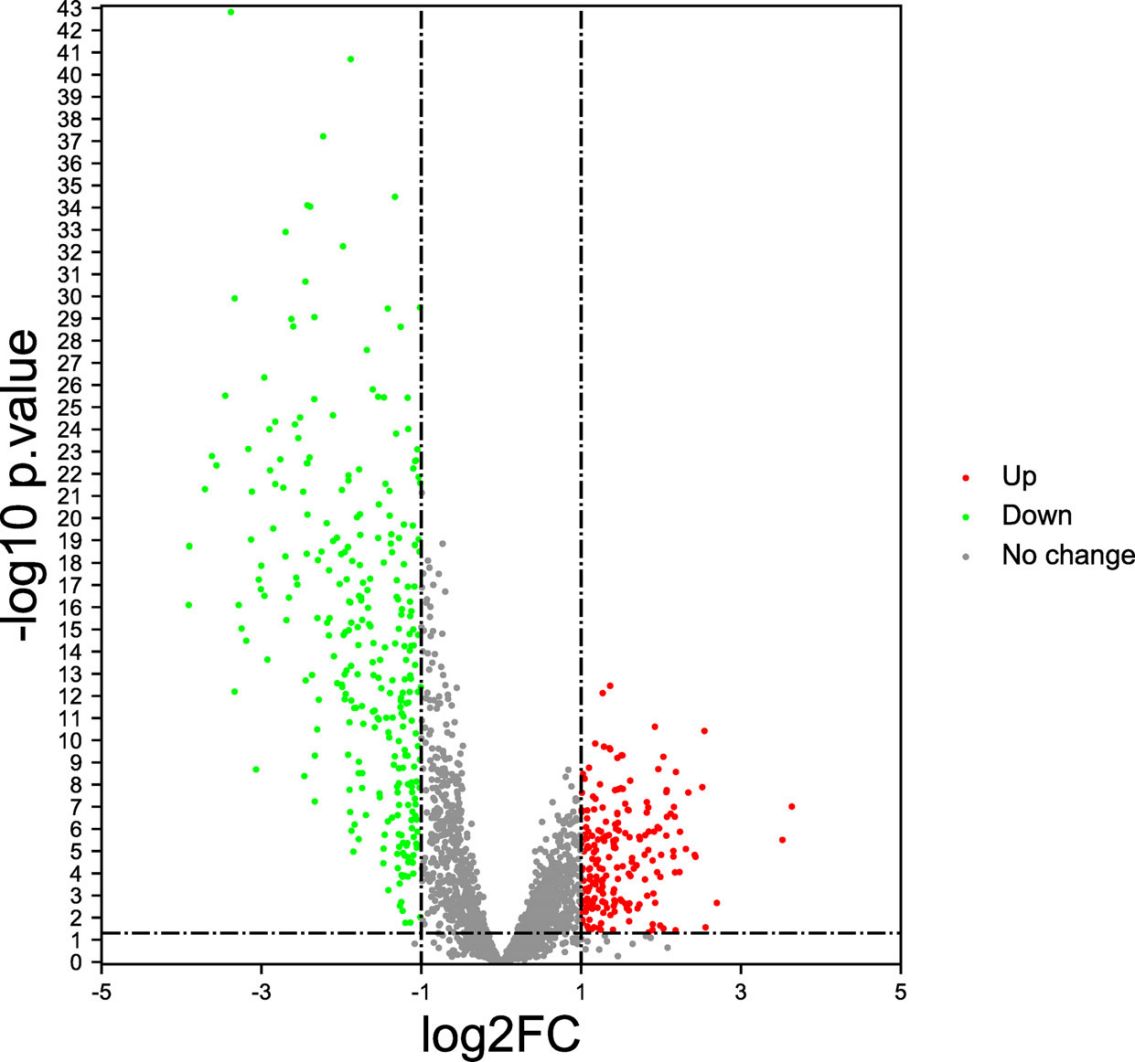
Immunoregulatory factors expression features (Volcano plot). The red dots represent significantly up-regulated, the green dots represent significantly down-regulated, and the gray dots represent no difference change.

In this study, by analyzing the TCGA database, the gene expression profiles of 782 immune regulatory factors were identified. We identified 234 differentially expressed immunoregulatory factors with the criteria of the absolute logarithmic 2-fold change (FC) >1and the adjusted *P*-value of LIMMA <0.05 in GC compared to normal gastric samples, including 132 immunoregulatory factors with up-regulation and 111 immunoregulatory factors with down-regulation (**Figure 1**).

### Bioinformatics Analysis of Differential Expression of Immunoregulatory Factors

**Figure 2:**
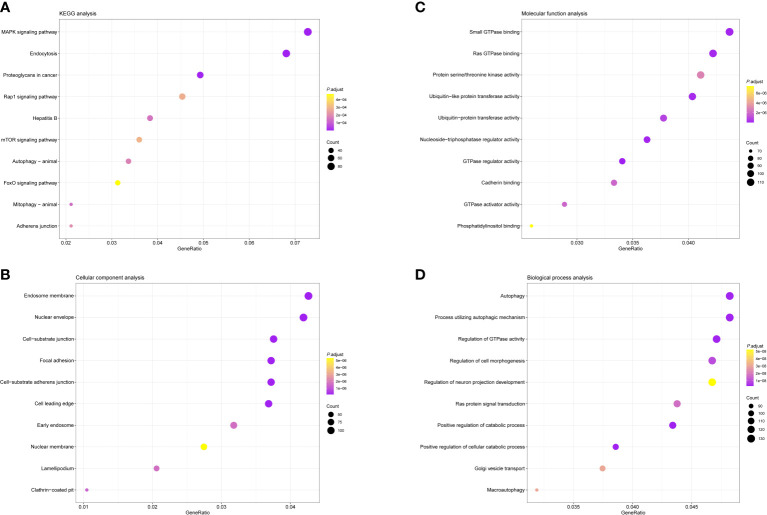
Bubble diagrams showing the enrichment analysis and signal pathway analysis results of Differential Expression of Immunoregulatory Factors. The top 10 enriched terms covering **(A)** BP, **(B)** MF and **(C)** CC are presented. **(D)** The top 10 enriched pathways of Differential Expression of Immunoregulatory Factors in KEGG analysis are introduced. GO, Gene Ontology; BP, biological processes; MF, molecular functions; CC, cellular components; KEGG, Kyoto Encyclopedia of Genes and Genomes.

Except for the regulation of immune response, we conducted GO and KEGG pathway analysis to evaluate the biological functions of these differently expressed immune regulatory factors. Enrichment of the KEGG pathway indicated that these differentially expressed immunoregulatory factors primarily took part in MAPK signaling pathway, endocytosis and proteoglycans in cancer (**Figure 2A**). GO CC analysis showed that these differentially expressed immunoregulatory factors were significantly enriched in endosome membrane, nuclear envelope, cell-substrate junction and focal adhesion (**Figure 2B**). For GO MF analysis, the first four significantly enriched terms are small GTPase binding, Ras GTPase binding, protein serine/threonine kinase activity, and ubiquitin-like protein transeferase activity (**Figure 2C**). The first four significantly richer BP terms included autophagy, a process utilizing autophagic mechanism, regulation of GTPase activity and regulation of cell morphogenesis (**Figure 2D**).

### The Prognostic Significance of Immunomodulatory Factors

**Figure 3:**
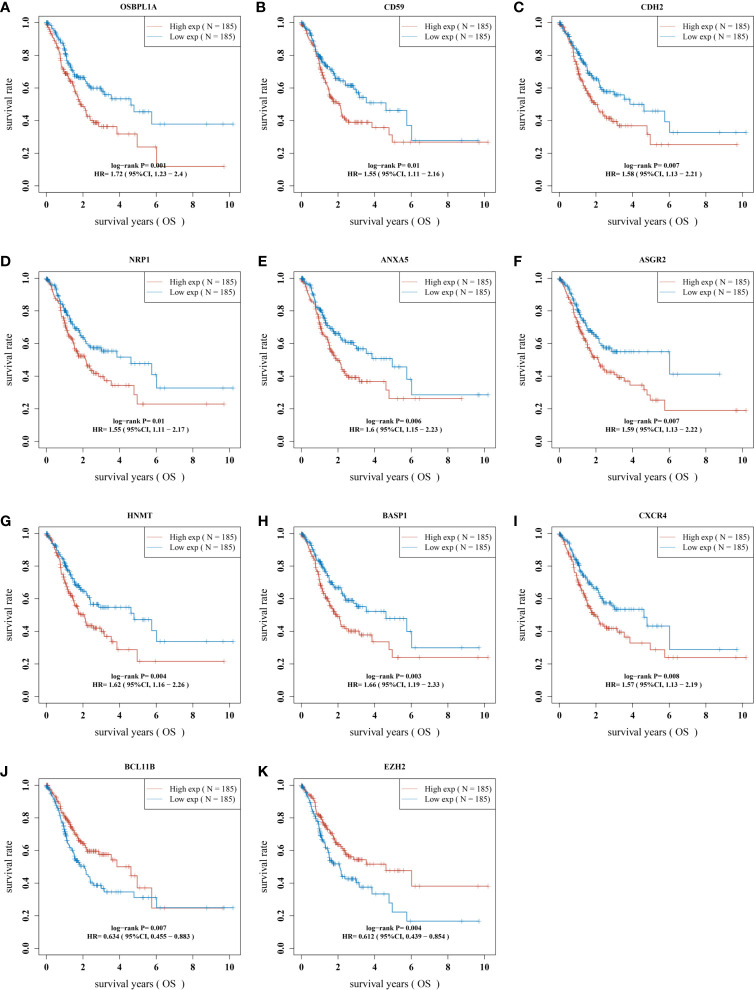
The prognostic value of immunomodulatory factors in GC. The correlation analysis between the expression levels of 11 immune regulatory factors and the OS of gastric cancer patients was analyzed, including **(A)** OSBPL1A, **(B)** CD59, **(C)**CDH2, **(D)** NRP1, **(E)** ANXA5, **(F)** ASGR2, **(G)** HNMT, **(H)** BASP1, **(I)** CXCR4, **(J)** BCL11B and **(K)** EZH2. OS, overall survival.

**Figure 4:**
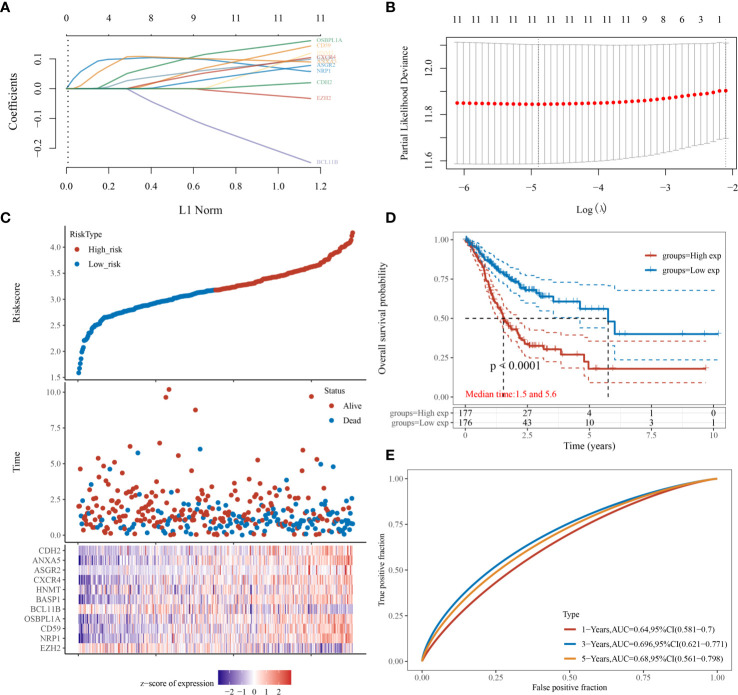
Prognostic significance analysis of immunomodulatory factor markers. **(A, B)** LASSO regression with tenfold cross-validation of 11. **(C)** survival of GC patients by the Signature risk score. **(D)** Kaplan–Meier analysis of high-risk group and low-risk group. **(E)** Time-dependent ROC at 1, 3 and 5-year area (middle curve of the AUC).

Then, we evaluated the significance of immunoregulatory factors on the prognosis of patients with GC. Univariate Cox regression and Kaplan–Meier analysis showed that higher expression of nine regulatory factors, including OsBPL1a, CD59, CDH2, NRP1, ANXA5, ASGR2, HNMT, BASP1, CXCR4, and were associated with lower survival rates of GC patients (**Figures 3A–I**). On the contrary, higher expression of EZH2 and BCL11B were associated with longer survival rates of GC patients (**Figures 3J, K**). We established a prognostic signal based on the multivariate Cox regression of ABCB6, FLVCR1, SLC48A1 and SLC7A11. Risk Score = (−0.028) ∗ of EZH2 + (0.06. 4) ∗ NRPl + (0.1308) ∗ CD59 + (0.15. 3) ∗ OsBPL1a + (−0.2268) ∗ BCL11B + (0.09 22 is) ∗ BASP1 + (0.0989) ∗ HNMT + (.0954) ∗ of CXCR4 + (0.0702) ∗ ASGR2+ (0.09 15) ∗ ANXA5+ (0.0168) ∗ CDH2. LASSO regression with tenfold cross-validation was performed to get the optimal lambda value that came from the minimum partial likelihood deviance, which was related to 11 genes that were significantly associated with OS (**Figures 4A, B**). **Figure 4C** shows that the survival of GC patients could be significantly predicted by the Signature risk score. Kaplan–Meier analysis revealed that the high-risk group presented dramatically shorter OS than the low-risk group (**Figure 4D**). Time-dependent ROC at 1, 3 and 5-year area (middle curve of the AUC) were 0.64, 0.696, and 0.68, respectively (**Figure 4E**).

### Analysis of the Correlation Between CXCR4 and Clinical Characteristics

**Figure 5:**
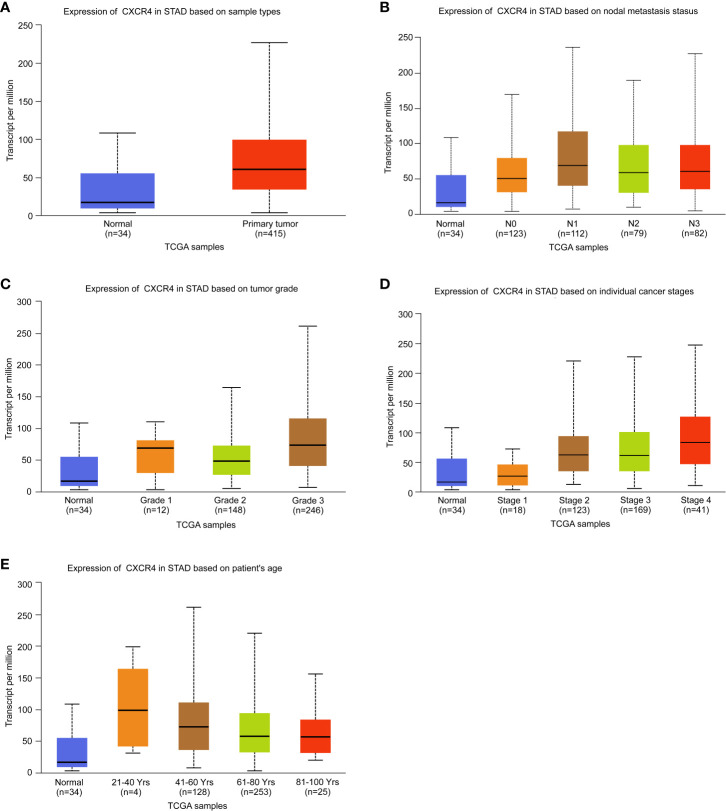
Analysis of the expression level of CXCR4 in GC. The expression level of CXCR4 was analyzed based on **(A)** sample type, **(B)** nodal metastasis status, **(C)** tumor grade, **(D)** individual cancer stage, and **(E)** patient age.

The above analysis revealed that CXCR4 was a key immunoregulatory factor in GC, so CXCR4 was selected for further analysis. According to the TCGA database, we found CXCR4 in GC was dramatically up-regulated in comparison with that in normal samples (**Figure 5A**). According to nodal metastasis status, stage, grade, stage and age, CXCR4 in GC was further analyzed. The results showed that CXCR4 was up-regulated in all N-stages of GC, with the strongest expression in N1 stage gastric cancer (**Figure 5B**). CXCR4 expression was positively related to the advanced stage and grade of GC. CXCR4 had the highest expression level in grade 3 and stage 4 samples, respectively (**Figures 5C, D**). Very interestingly, the CXCR4 expression level was negatively correlated to the age of patients with GC (**Figure 5E**).

**Figure 6:**
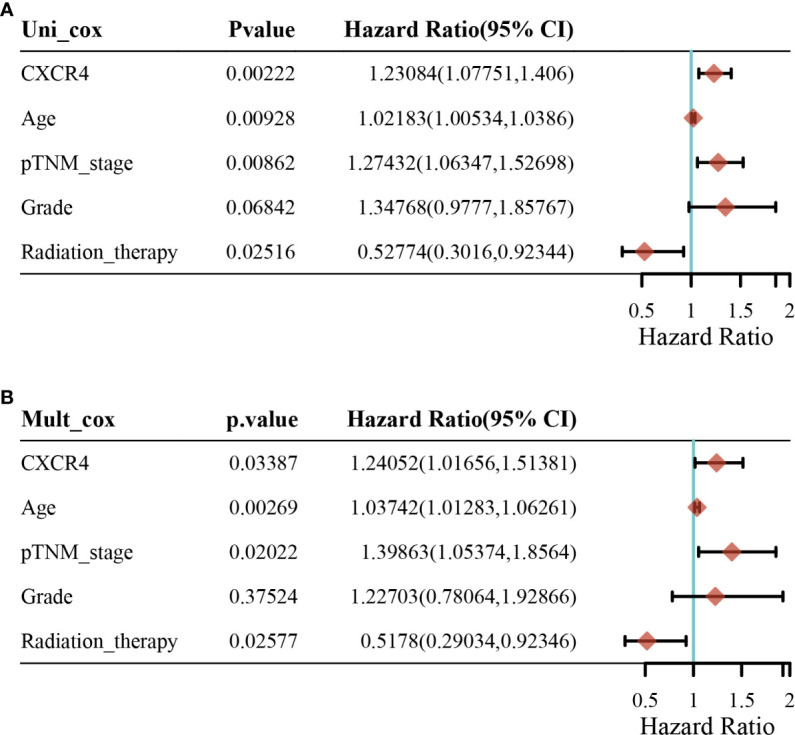
Univariate analysis and multivariate analysis for patients with gastric cancer. **(A)** Univariate analysis and **(B)** multivariate analysis.

**Figure 7:**
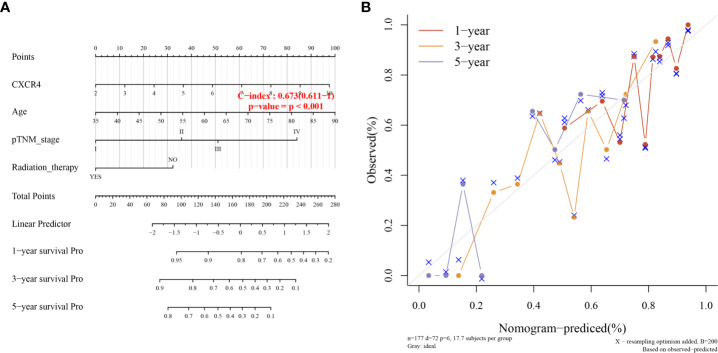
Clinical association analysis and Fitting analysis**. (A)** Association Analysis between survival probability and risk score. **(B)** Time Fitting analysis.

In addition, univariate analysis (**Figure 6A**) and multivariate analysis (**Figure 6B**) indicated that CXCR4 was an independent prognostic indicator for GC patients in TCGA. Then, we based on AJCC stage and CXCR4 multivariate expressed Cox coefficient regression model constructed nomogram, and 1 year by AJCC calculated a score for each patient stage variable value, so as to arrive GC. The patient’s 3- and 5-year survival probability and risk score (**Figure 7A**). Next, through the evaluation of the C index and AUC value, as well as the evaluation of the discriminant efficiency and prediction accuracy of the nomogram in the training set. Our results show that the nomogram is well-calibrated because the curve is close to the diagonal (**Figure 7B**).

**Figure 8:**
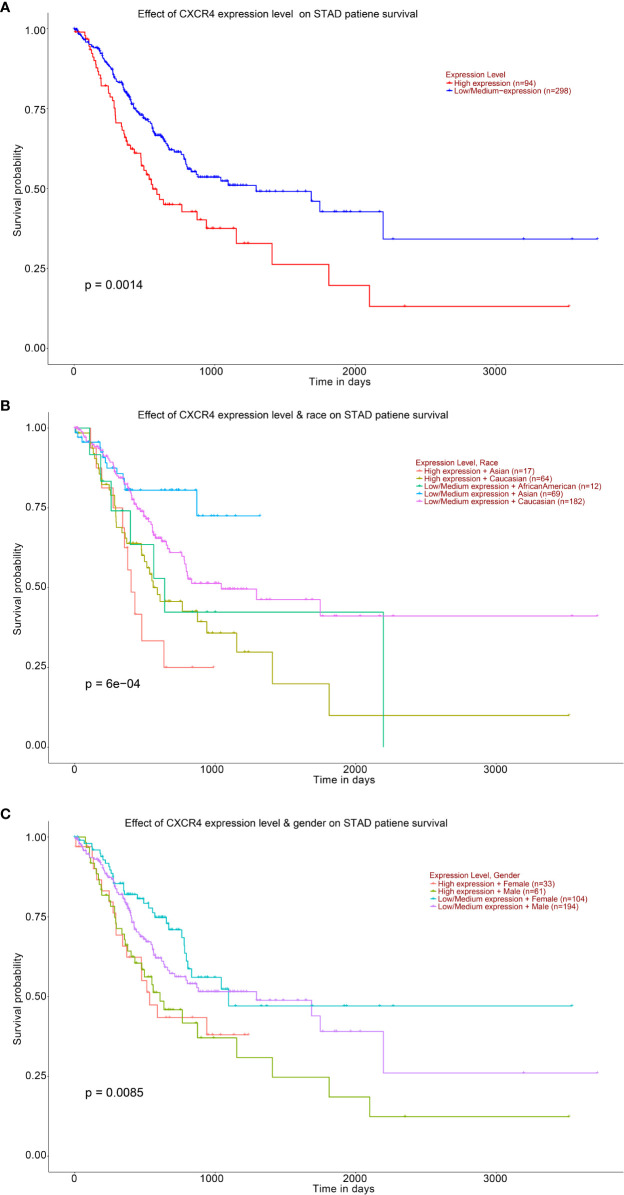
Analysis of the expression level of CXCR4 and the ten-year survival of STAD patients. Kaplan–Meier based on **(A)** STAD patient, **(B)** STAD patient’s race, **(C)** STAD patient’s gender.

Survival analysis showed that STAD patients with higher levels of CXCR4 had lower survival (**Figure 8A**). Compared with Caucasians with a higher level of CXCR4, Asians with a higher level of CXCR4 had lower survival (**Figure 8B**). Compared with females with lower levels of CXCR4, males with lower levels of CXCR4 had lower survival (**Figure 8C**). In general, up-regulated CXCR4 in GC exhibits a close relationship to GC occurrence and development.

### CXCR4 Promoted GC Cells Proliferation

**Figure 9:**
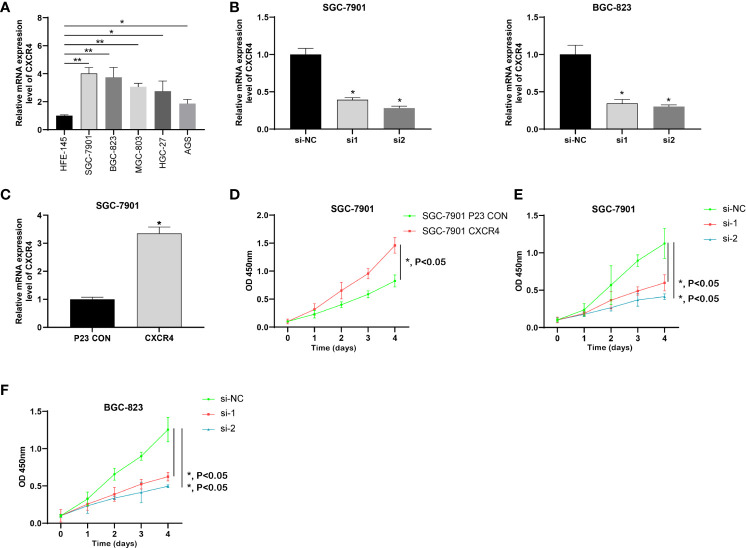
CXCR4 was up-regulated in GC and promoted the proliferation of GC cells. **(A)** The expression of CXCR4 in GC cells was determined by RT-qPCR. **(B)** The specific siRNA knockdown the expression of CXCR4 in the GC cell line. **(C)** The CXCR4 overexpression vector increase the expression of CXCR4 in the GC cell line. **(D)** Overexpression of CXCR4 promoted the proliferation of the SGC-7910 cell line. Knockdown of CXCR4 inhibited the proliferation of SGC-7910 **(E)** and BGX-823 **(F)** cell lines. * means P < 0.05; ** means P < 0.01.

We analyzed the expression level of CXCR4 in five GC cell lines, and the results showed that CXCR4 was significantly up-regulated in GC cell lines, especially SGC-7901 and BGC-823 (**Figure 9A**). We designed CXCR4 siRNA to further investigate CXCR4 function in GC cells. We established the CXCR4 knockdown cell line in SGC7901 and AGS cells, and its knockdown efficiency was detected by RT-qPCR (**Figure 9B**). RT-qPCR showed that in infected SNHG16 cells, the expression of SNHG16 in SGC-7901 cells was significantly increased (**Figure 9C**). Compared to control cells, two siRNAs could effectively knock down CXCR4 in two cells. Overexpression of CXCR4 significantly promoted GC cell proliferation (**Figure 9D**). Abated CXCR4 dramatically inhibited GC cell proliferation (**Figures 9E, F**). Collectively, CXCR4 was a promoter in facilitating GC cell proliferation.

### CXCR4 Facilitated Cell Migration and Invasion of GC

**Figure 10:**
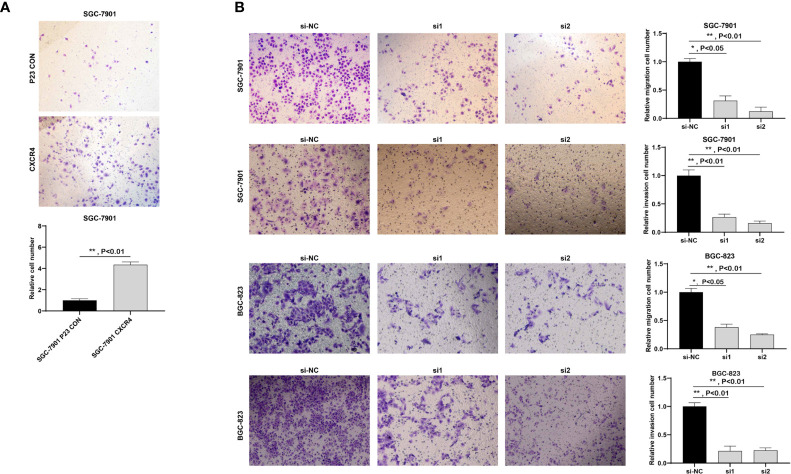
CXCR4 promoted the migration and invasion of GC cells. **(A)** Overexpression of CXCR4 promoted metastasis of the SGC-7901 cell line. **(B)** Knockdown of CXCR4 inhibited the migration and invasion of SGC-7901 and BGC-823 cell lines.

Since GC is highly malignant, it is prone to multiple metastases in the early stage and the survival rate is extremely low. Here, we studied the metastasis of GC. Because CXCR4 exerted an effect on GC cell proliferation, we will explore the influence of CXCR4 on GC cell invasion. We conducted Transwell analysis to detect cell invasion capability. Overexpression of CXCR4 in SGC7901 cells the invasion ability was greatly promoted (**Figure 10A**). After knocking down CXCR4 in SGC7901 and AGS cells, the invasion ability was greatly inhibited (**Figure 10B**).

## Discussion

Abnormally expressed immunoregulatory factors are associated with a variety of malignant behaviors in multiple types of carcinoma. A series of immunoregulatory factors are shown to play vital parts in GC. For example, a higher level of soluble PD-L1 (sPD-L1) in plasma predicts shorter overall survival for GC patients (29, 30). Wang et al. showed that signals including 8-immune-related genes (IRG) could function as a predictor of the OS rate of GC patients and their response to immune checkpoint inhibitors (28). Additionally, a prognostic model with three immune-related genes (SEMA6A, LTBP1 and BACH2) could predict the OS rate of GC patients with different microsatellite instability states. Here, we evaluated the expression patterns of 782 immune regulatory factors in GC and determined that 234 immune regulatory factors were significantly dysregulated in GC compared to the normal sample. In addition, except for immune regulation, we also found that these dysregulated immune regulatory factors were related to the MAPK signaling pathway, endosome membrane, small GTPase binding and autophagy. This indicated that they may have multiple key roles in GC. Finally, we found that the imbalance of 11 immune regulatory factors could predict the overall survival time of gastric cancer, including EZH2, NRP1, CD59, OsBPL1aBCL11B, BASP1, HNMT, CXCR4, ASGR2, ANXA5, CDH2. This study shows for the first time that immunomodulatory factors might be utilized as potential biomarkers for GC prognosis.

In the past few decades, people have made a lot of efforts to uncover potential indicators for GC’s prognosis. For instance, PFKFB4 is a promising biomarker for predicting the poorly prognostic status of GC patients (31). Overexpressed CLC-3 is an indicator for poorly prognostic status of GC. The overexpression of CLC-3 is regulated by XRCC5, which is a biomarker for the poor prognosis of GC (32). Nevertheless, the 5-year survival rate of distant GC is still as low as 6%. Therefore, there is an urgent need to find new biomarkers. Here, we are trying to construct a signal based on immune regulatory factors to make predictions. We made a distinction between the prognostic risk signals with 11 genes, including EZH2, NRP1, CD59, OsBPL1A, BCL11B, BASP1, HNMT, CXCR4, ASGR2, ANXA5CDH2. It is worth noting that compared to previously reported prognostic indicators (T, N, M clinical stage), our prognostic risk characteristics present higher accuracy, with AUC value >0.8. To sum up, our findings show that the risk signal could be utilized as potential biomarkers, providing more clinical applications and effective treatment guidelines.

Immune regulatory factors may also be related to tumor progression except for the prognostic value of risk signals. EZH2 (Enhancer of Zeste homolog 2) belongs to a member of the Polycomb gene family and is an important class of epigenetic modulators in inhibiting transcription (33). Polycomb suppression complex 2 (PRC2) is one core complex of PCG, mediating gene silencing mainly *via* modulating chromatin structure (34). As the enzymatic subunit of PRC2, EZH2 alters gene expression *via* trimethylating Lys-27 in histone 3 (H3K27me3) (33, 35). H3K27Me3 is reported to be related to the inhibition of gene expression and is considered to be a key epigenetic event in the development of tissues and the determination of stem cell fate. In GC, inhibiting EZH2 and EGFR exerts a synergistic effect on cell apoptosis *via* raising autophagy in GC cells (36). EZH2 mediates the promotion of 5-FU resistance in GC by epigenetically inhibiting FBXO32 expression (37). EZH2 induces the transition of epithelial–mesenchymal and pluripotency phenotype of GC cells *via* combination with the PTEN promoter (38). CD59 is a glycosylphosphatidylinositol-anchored membrane protein, acting as a suppressor of membrane attack complex to modulate complement activation (39). Current reports have revealed high expression of CD59 in various cell lines and tissues of cancer. It is found that CD59 is necessary for the epithelial cancer stem cells to evade complement monitoring. In breast cancer, CD59 could promote the growth of neoplasm and predict the poorly prognostic status (40). The transcription factor BCL11B is an important immunoregulatory factor that can promote the typical and adaptive differentiation of NK cells (41). Emerging reports have shown that BASP1 could modulate multiple biological behaviors, such as cell proliferation, apoptosis, and differentiation (42, 43). More and more pieces of evidence confirm that BASP1 plays as a potential suppressor of tumor and functions importantly in various carcinomas, including thyroid carcinoma (44), stomach carcinoma (45) and lung carcinoma (46). Nevertheless, it is unclear the influence of BASP1 on GC. In GC, BASP1 suppressed cell growth and metastasis *via* inhibiting the Wnt/β-catenin pathway (45). This study confirms for the first time that the imbalance of these immune regulatory factors is associated with the survival time of GC patients.

CXCR4 displays a key role in a variety of cancers. CXCR4 expression in cancer cells is negatively related to the prognosis of the disease and serves as an independent factor of other prognostic parameters. The discovery involves tumor-initiating cancer stem cells (CSC) of CXCR4 expression which is conducive to CXCR4 in resistance to treatment, recurrence, metastasis and poor clinical outcome. The CXCR4/RhoA signaling pathway participates in miR-128-modulated human thyroid carcinoma cells proliferation and apoptosis (47). In endometrial cancer, the CXCL12/CXCR4 axis induces proliferation and invasion (48). Recently, some studies have revealed the function of CXCR4 in GC. For example, the block of CXCR4/mTOR signaling pathway induces anti-metastatic properties and autophagic cell death of CER cells in disseminated peritoneal GC (49). Here, we systematically investigate the expression features, possible effects and mechanisms of CXCR4 in GC. We discover CXCR4 is highly expressed in GC and closely related to the prognosis of GC. Reducing CXCR4 largely hinders GC cells proliferation, migration and invasion *in vitro*. These results demonstrated that CXCR4 acted as an oncogene and is a potential biomarker for GC treatment.

There are several limitations that should be taken into consideration. First of all, this is a bioinformatics analysis based on public databases. Therefore, the functions of the three new immunomodulatory genes (BASP1, OsbPL1A and CD59) need to be further explored. At the same time, we have not verified the expression of key immune regulatory genes in clinical samples. Therefore, we plan to continue to collect patient and clinical data to further verify this issue in the future. Finally, we will further verify the results of CXCR4 *in vitro* studies through an animal model assay.

## Conclusion

In conclusion, this study analyzed and constructed a gastric cancer prognosis model based on the expression profile of immunoregulatory factor-related genes, which provided new information for gastric cancer research. We identified 234 differently expressed immunoregulatory factors and established risk signals formed by 11immunoregulatory factors for prognostic evaluation of gastric cancer. SED was performed on the TCGA data set. Finally, we focus on CXCR4 expression and find that CXCR4 is greatly up-regulated in GC. Additionally, we discover CXCR4 is an oncogene of GC cell proliferation, migration and invasion. Our research provides a new biomarker-based on immunomodulatory factor analysis for GC prognosis and treatment.

## Data Availability Statement

The datasets presented in this study can be found in online repositories. The names of the repository/repositories and accession number(s) can be found below: https://cancergenome.nih.gov/, TCGA.

## Author Contributions

Conception and design: SX and FL. Development of methodology: SX, CW, HL and YH. Sample collection: FL, XZ and LF. Analysis and interpretation of data: YQ, JJ and ZQ. Writing, review, and/or revision of the manuscript: MM, SB, SX, JJ, and LF.

## Conflict of Interest

The authors declare that the research was conducted in the absence of any commercial or financial relationships that could be construed as a potential conflict of interest.
